# Dynamic Arterial Elastance as a Predictor of Intraoperative Fluid Responsiveness in Elderly Patient over 70 Years of Age Undergoing Spine Surgery in the Prone Position Under General Anesthesia: A Validation Study

**DOI:** 10.3390/jcm14041247

**Published:** 2025-02-13

**Authors:** Eun Jung Oh, Eun Ah Cho, Joohyun Jun, Sung Hyun Lee, Seunghyeon Lee, Jin Hee Ahn

**Affiliations:** Department of Anesthesiology and Pain Medicine, Kangbuk Samsung Medical Center, Sungkyunkwan University School of Medicine, Seoul 03063, Republic of Korea; angy02@naver.com (E.J.O.); eunah84.cho@samsung.com (E.A.C.); joohyun.jeon@samsung.com (J.J.); 4321hoho@naver.com (S.H.L.); seunghyeon86.lee@samsung.com (S.L.)

**Keywords:** general anesthesia, fluid responsiveness, arterial elastance

## Abstract

**Background**: Optimizing fluid therapy is critical for maintaining hemodynamic stability in elderly patients undergoing major surgeries. Dynamic arterial elastance (Eadyn), defined as the ratio of pulse pressure variation (PPV) to stroke volume variation (SVV), has been proposed as a predictor of fluid responsiveness, especially in challenging conditions like prone-positioned spine surgery under general anesthesia. **Methods:** Hemodynamic parameters were measured before and after fluid loading with 500 mL of crystalloid solution. Patients were classified as responders or non-responders based on a ≥15% increase in mean arterial pressure (MAP) post-fluid administration. Predictive performance of these parameters was assessed using receiver operating characteristic (ROC) analysis. **Results:** Of the 37 patients, 15 were classified as responders and 22 as non-responders. Eadyn demonstrated poor predictive performance (AUC = 0.508). In contrast, SVV (AUC = 0.808), PPV (AUC = 0.738), and C (AUC = 0.741) exhibited moderate to high predictive ability. Responders exhibited significantly higher baseline SVV, PPV, and net arterial compliance compared to non-responders. **Conclusions:** Dynamic arterial elastance (Eadyn) showed limited predictive ability for fluid responsiveness in elderly patients undergoing spine surgery in the prone position. In contrast, stroke volume variation (SVV), pulse pressure variation (PPV), and net arterial compliance (C) demonstrated superior reliability, with SVV emerging as the most accurate predictor.

## 1. Introduction

Optimizing fluid therapy is essential for hemodynamic management during the perioperative period, particularly in major surgeries. A sustained drop in arterial pressure reduces tissue perfusion, potentially leading to organ dysfunction and death [1,2,3]. While fluid administration is the primary treatment for hypotension, the assumption that increasing stroke volume (SV) will raise arterial pressure is not always reliable due to variations in arterial tone [4]. Dynamic arterial elastance (Eadyn), the ratio of pulse pressure variation (PPV) to stroke volume variation (SVV), provides a real-time assessment of arterial tone, a critical determinant in fluid management. By incorporating both PPV and SVV, Eadyn can predict how changes in SV may influence arterial pressure, particularly under varying arterial tones [5,6].

Previous studies have demonstrated that PPV and SVV are highly predictive of fluid responsiveness in both supine and prone surgical positions, with AUROC values over 0.9 [7,8]. Despite this strong predictive performance, these parameters are significantly influenced by intrinsic factors such as lung–heart interactions and vascular conditions [9]. While their predictive value has been well established, the ability of Eadyn, as a ratio of PPV to SVV, to predict fluid responsiveness in prone surgical settings remains underexplored.

In elderly patients, who often present with reduced physiological reserve, intraoperative fluid management becomes more complex. The prone position, commonly used in spine surgeries, alters hemodynamics by affecting venous return, intra-abdominal pressure, and pulmonary mechanics [7,8]. Furthermore, elderly patients often have altered arterial tone and increased arterial stiffness, which may affect their responsiveness to fluid administration [10,11]. Assessing fluid responsiveness in elderly patients presents significant challenges due to their altered physiological reserve and vascular properties.

Considering the combined challenges of aging and the prone surgical position, predicting fluid responsiveness in elderly patients is critical for anesthesiologists, and the ability to predict fluid responsiveness using Eadyn in elderly patients could offer valuable insights for intraoperative management [8,12]. Therefore, the aim of this study is to assess whether dynamic arterial elastance (Eadyn) can predict fluid responsiveness in elderly patients over the age of 70 undergoing spine surgery in the prone position under general anesthesia.

## 2. Materials and Methods

### 2.1. Study Population and Ethics

This prospective study was approved by the Ethics Board of the Kangbuk Samsung Hospital Institutional Review Board, Seoul, Republic of Korea (approval number: KBSMC 2021-07-073, dated 2 November 2021). Prior to patient enrollment, the study was registered at https://cris.nih.go.kr under registration number KCT 0007032 (registration date: 23 February 2022). The study commenced after obtaining informed consent from all participants and was conducted at a single tertiary hospital in accordance with the principles of the Declaration of Helsinki. This manuscript utilized OpenAI’s ChatGPT(Version 4o) to assist with language editing and sentence structuring. The authors take full responsibility for the accuracy and scientific validity of the content presented

Inclusion criteria were patients aged 70 years or older classified as ASA class I-IV, undergoing spine surgery in the prone position under general anesthesia. Eligible patients exhibited at least one of the following clinical signs of hypotension or preload dependency after anesthesia induction: mean arterial pressure (MAP) < 65 mmHg, systolic blood pressure < 90 mmHg or a decrease in systolic blood pressure by more than 40 mmHg from baseline, or stroke volume variation (SVV) > 10%. Exclusion criteria included patients younger than 70 years, those with radial artery vascular abnormalities, body mass index (BMI) greater than 30 kg/m^2^ or less than 15 kg/m^2^, a history of heart disease (such as valvular disease, left ventricular ejection fraction (LVEF) < 50%, or arrhythmia), pulmonary disease, or kidney disease. Patients who could not undergo radial artery catheterization due to conditions such as end-stage renal disease on dialysis or scheduled for dialysis, or a history of breast cancer surgery, were also excluded. Additional exclusion criteria included patients with unstable hemodynamic status or those undergoing emergency surgery.

### 2.2. Anesthetic Management and Prone Positioning

The anesthesia protocol followed the standard general anesthesia guidelines of our institution. No premedication was administered. Before the induction of general anesthesia, standard monitoring, including electrocardiography, pulse oximetry, non-invasive blood pressure, BIS, and train-of-four (TOF), was initiated. Invasive arterial monitoring was performed using an arterial line placed while the patient was awake just before anesthesia. A 22G angiocatheter was inserted into either the right or left radial artery following local infiltration with 0.5 mL of 1% lidocaine. Intravenous administration of 1.5 mg/kg of 1% propofol was performed for induction. Upon confirming loss of consciousness, TOF was calibrated and measured every 5 min. Simultaneously, 8% sevoflurane and 0.8 mg/kg rocuronium were administered. Intubation was performed after 90 s using an endotracheal tube. Anesthesia was maintained with sevoflurane, titrated to maintain a BIS value between 40 and 60. Mechanical ventilation settings included a fresh gas flow of 3 L/min and a tidal volume of 8 mL/kg. After the induction of anesthesia was completed, the patient was positioned in the prone position. The patient was placed horizontally, face-down, with rolls positioned bilaterally to support the chest wall. Once this position was secured, the patient’s head was rotated to either the left or right and placed on a U-shaped pad for support. The arms were positioned on padded arm boards on both sides of the head. Hip flexion was adjusted to an angle between 10° and 20°, and protective padding was used to support knee flexion, set between 100° and 120°.

### 2.3. Arterial Pressure Monitoring and Arterial Tone Parameters

The angiocatheter was connected to a pressure transducer (Acumen IQTM, Edwards Lifesciences, Saint Ana, CA, USA) and linked to the HemosphereTM monitor (Edwards Lifesciences). After zeroing at the mid-axillary level, the following parameters were obtained: mean arterial pressure (MAP), heart rate (HR), cardiac output (CO), stroke volume (SV), stroke volume index (SVI), pulse pressure variation (PPV), stroke volume variation (SVV), and dynamic arterial elastance (Eadyn). The ACUMEN sensor calculates stroke volume (SV) and cardiac output (CO) in real-time through arterial waveform analysis, utilizing the pulse contour analysis technique. This method estimates SV and CO by analyzing the morphology and amplitude of the arterial pressure waveform. Other arterial tone parameters were calculated as each formula. The static component reflects a unique pressure–volume (P–V) relationship and can be characterized by two elements: a resistive element, represented by systemic vascular resistance (SVR = MAP/CO × 80), and a pulsatile element, represented by net arterial compliance (C = SV/arterial pulse pressure). [13] This distinction arises from the oscillatory nature of arterial flow and the mechanical properties of the arterial system. Effective arterial elastance (Ea = 0.9 × SAP/SV) is an integrative variable that captures both the steady and pulsatile components of arterial load. [14] All patients were studied after a 2- to 3-min period of hemodynamic stability, during which there were no changes in the anesthetic protocol, ventilator settings. Baseline hemodynamic measurements were taken, followed by intravenous fluid loading with 500 mL of crystalloid (plasma solution, HK Inno-n, Cheongju, Republic of Korea) administered over 10–20 min. [5] Hemodynamic measurements were then performed 2–5 min after fluid loading.

### 2.4. Responders Versus Non-Responders

Patients were classified based on the increase in MAP after VE into MAP responders (≥15%) and MAP non-responders (<15%). This threshold was chosen under the assumption of a perfect 1:1 arterial pressure–flow coupling and optimal mechanical efficiency, where a 15% increase in SV should correspondingly increase MAP by 15% [6,15,16].

### 2.5. Statistical Analysis

The sample size calculation was performed to achieve 80% power. It was estimated based on a diagnostic accuracy (area under the ROC curve) of 0.9, with the null hypothesis set at 0.7. To detect a difference of 0.2 between the AUCs, a sample size of 42 participants was determined to be necessary. The normality of the data distribution was tested using D’Agostino-Pearson test. Data are presented as mean (±standard deviation), median (interquartile range), numbers (n), and percentages (%). Continuous variables were compared using the *t*-test or Mann–Whitney U test, as appropriate, and the Shapiro–Wilk test was used to explore normality. Categorical variables were compared using Pearson’s chi-square or Fisher’s exact tests. Differences between groups were also compared using an independent samples *t*-test, while comparisons between pre-loading and post-loading periods were analyzed using a paired *t*-test for repeated measures. All statistical analyses were performed using MedCalc^®^ Statistical Software version 20.014 (MedCalc Software Ltd., Ostend, Belgium; https://www.medcalc.org accessed on 31 October 2024) and R, version 4.1.2 (The R Foundation). We used 2-tailed tests in all analyses, with *p* values < 0.05 considered statistically significant.

## 3. Results

A total of 43 patients were assessed for eligibility. Of these, six were excluded due to refusal to participate (*n* = 3), connection errors (*n* = 2), and arrhythmia (*n* = 1). Consequently, 37 patients were included in this study, with 15 classified as responders and 22 as non-responders based on their mean arterial pressure (MAP) increase after fluid loading (Supplementary Figure S1). Table 1 summarizes the patient characteristics, showing no significant differences between the two groups in terms of gender, age, weight, height, BMI, ASA physical status, or ventilator settings.

### 3.1. Hemodynamic Changes

**Figure 1:**
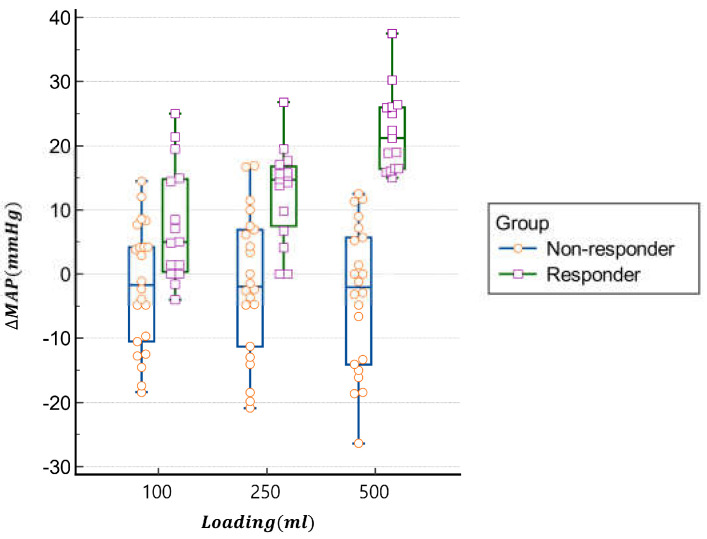
Individual values in responder (*n* = 15) and non-responders (*n* = 22) of variations MAP after fluid loading.

Figure 1 shows the individual values of MAP trends in responders (*n* = 15) and non-responders (*n* = 22). Table 2 presents the hemodynamic changes observed before and after fluid loading. In responders, MAP significantly increased from 70 ± 9 mmHg to 85 ± 10 mmHg (*p* < 0.0001), whereas no significant MAP increase was observed in non-responders (80 ± 12 mmHg to 76 ± 12 mmHg, *p* = 0.0512). Heart rate decreased significantly in both groups post-loading, but the intergroup difference was not statistically significant. Both groups experienced a significant increase in stroke volume (SV) and stroke volume index (SVI) after fluid loading, but the changes were comparable between responders and non-responders.

Following fluid loading, both pulse pressure variation (PPV) and stroke volume variation (SVV), indicators of dynamic arterial tone, showed a significant decrease in both responders and non-responders. However, the initial pre-loading values of PPV (20 ± 3% vs. 15 ± 7%, *p* = 0.0187) and SVV (17 ± 4% vs. 12 ± 5%, *p* = 0.0030) were significantly higher in responders compared to non-responders, indicating greater fluid responsiveness in the former group.

### 3.2. Arterial Tone Parameters

**Figure 2:**
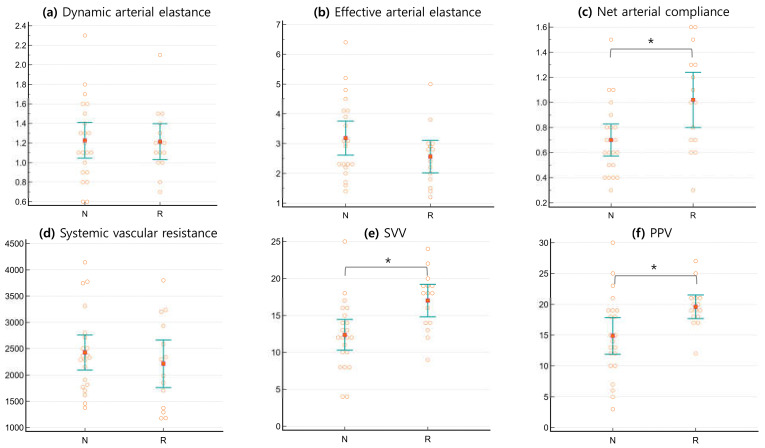
Comparison of the distribution of baseline arterial tone parameters between responders and non-responders at pre-loading phase. (**a**) Eadyn, dynamic arterial elastance; (**b**) Ea, effective arterial elastance; (**c**) C, net arterial compliance; (**d**) SVR, systemic vascular resistance; (**e**) SVV, stroke volume variation; (**f**) PPV, pulse pressure variation). Asterisk signs (*) indicate statistically significant differences between responders and non-responders for each variable. The orange square represents the median, while the green bar indicates the 95% confidence interval (CI).

The effect of fluid loading on arterial tone parameters was shown in Table 3 and Figure 2. The pre-loading values of dynamic arterial elastance (Eadyn), effective arterial elastance (Ea), and systemic vascular resistance (SVR) did not differ significantly between the two groups. However, net arterial compliance (C) was significantly higher in responders (1.02 ± 0.40 versus 0.70 ± 0.29, *p* = 0.0076). Additionally, after fluid loading, the responder group showed no significant changes in arterial tone parameters (Eadyn, Ea, C, and SVR), whereas the non-responder group exhibited statistically significant increases (C) or decreases (Eadyn, Ea, and SVR) in all parameters.

### 3.3. Receiver Operating Characteristic (ROC) Analysis

**Figure 3:**
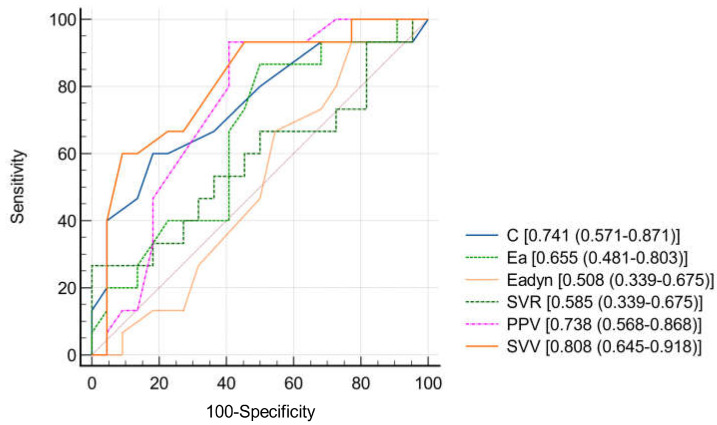
Comparison of receiver operating characteristics curves regarding the ability of studied arterial tone parameters to discriminate MAP responder patients (MAP increase ≥ 15%) and MAP non-responder patients after volume loading.

Figure 3 presents the receiver operating characteristic (ROC) curves comparing the ability of arterial tone parameters (including PPV and SVV) to discriminate between MAP responders and non-responders. The AUC values for Eadyn, C, Ea, SVR, PPV, and SVV were 0.508, 0.741, 0.655, 0.585, 0.738, and 0.808, respectively. During the fluid pre-loading phase, Eadyn demonstrated poor predictive performance, while C, PPV, and SVV showed moderate or higher prediction performance. Of these, SVV was most reliable predictors of fluid responsiveness.

## 4. Discussion

This study aimed to validate dynamic arterial elastance (Eadyn) as a predictor of intraoperative fluid responsiveness in elderly patients over 70 undergoing spine surgery in the prone position. The findings reveal that Eadyn alone showed poor predictive performance for fluid responsiveness, while other parameters, particularly stroke volume variation (SVV), pulse pressure variation (PPV), and net arterial compliance (C), demonstrated moderate or better predictive ability. These insights contribute to understanding the complex interplay of arterial tone and hemodynamic responsiveness in elderly patients in challenging surgical positions, emphasizing the limitations and benefits of using Eadyn in fluid management.

Previous studies have highlighted the utility of dynamic arterial elastance (Eadyn) in predicting arterial pressure responses to volume expansion (VE), suggesting that higher pre-infusion Eadyn values correlate with greater improvements in arterial pressure and advocating its clinical relevance in hemodynamic management. An Eadyn value above 0.7 is a strong predictor of pressure responsiveness with high sensitivity and specificity [5,6,17,18,19]. However, our study demonstrated that, although Eadyn did not perform effectively as a standalone predictor, other arterial tone indicators such as C, PPV, and SVV performed at a moderate or higher predictive level. The highest AUC was observed in SVV, which underscores its value as a predictive tool for fluid responsiveness in this patient population. This finding aligns with prior studies suggesting that SVV, as a dynamic parameter, can be particularly sensitive to preload responsiveness in elderly patients with altered vascular compliance and decreased arterial tone [20,21].

In contrast, Eadyn’s limited performance could be attributed to the baseline alterations in arterial compliance often observed in older adults, as well as the hemodynamic shifts induced by prone positioning, which may attenuate Eadyn’s sensitivity as a fluid responsiveness predictor. The prone position alters venous return, increases intra-abdominal pressure, and affects pulmonary mechanics, all of which influence preload and vascular compliance [7,22,23]. These changes may dampen the accuracy of Eadyn, as it depends on the interplay between SVV and PPV, both of which are influenced by mechanical ventilation and intrathoracic pressure variations in a prone position [23]. Furthermore, the combination of prone positioning and advanced age introduces additional hemodynamic challenges, as elderly patients are more likely to exhibit vascular stiffness and impaired compliance. While chronological aging was used as a practical threshold to identify this patient population, it is essential to acknowledge that biological aging may more closely correlate with the hemodynamic adaptations seen during surgery [24,25]. Understanding the interaction between prone positioning, arterial stiffness, and fluid responsiveness could inform tailored management strategies for this high-risk group. Expanding on these hemodynamic complexities will be critical for better understanding Eadyn’s limitations in such scenarios.

An interesting finding from our study is that the net arterial compliance value during the preloading phase was significantly higher in the responder group, and its predictive power for fluid responsiveness was excellent, with an AUC of 0.741. Net arterial compliance outperforms Eadyn in predicting fluid responsiveness in the context of age-related arterial stiffness and the prone position because it is less influenced by dynamic changes in the preload, more directly linked to intrinsic arterial properties, and accounts for pulsatile load and ventricular–arterial coupling [13]. However, the findings show the clinical utility of arterial compliance as a key determinant in optimizing fluid management strategies, particularly in high-risk populations. Clinically, incorporating net arterial compliance into hemodynamic assessments may help anesthesiologists better tailor fluid administration, especially in elderly patients undergoing prone-position surgeries. By identifying patients with higher baseline compliance, clinicians can predict a more favorable response to fluid loading, potentially improving intraoperative outcomes. Further studies are needed to explore the integration of arterial tone parameters like net arterial compliance into routine perioperative management across diverse surgical settings.

In this study, responders exhibited significantly higher baseline values of PPV, SVV, and net arterial compliance compared to non-responders. These findings suggest that baseline hemodynamic parameters may reflect a greater sensitivity to preload changes, indicating a higher likelihood of fluid responsiveness. The elevated baseline PPV and SVV in responders align with the principles of dynamic indices, which are known to be influenced by the magnitude of respiratory-induced variations in preload and cardiac output [26]. This could signify that responders were operating closer to the steeper portion of the Frank–Starling curve, where fluid administration would more effectively augment stroke volume [27]. Additionally, the higher baseline net arterial compliance in responders may indicate more preserved arterial elasticity despite age-related vascular changes. Aging is associated with reduced arterial compliance, increased arterial stiffness, and impaired ventricular–arterial coupling, all of which impact the cardiovascular system’s adaptability to preload changes. In responders, the preservation of baseline compliance may allow for more effective hemodynamic adjustments in response to fluid loading, contributing to improved stroke volume and arterial pressure outcomes. Understanding how these age-related changes influence both arterial tone and dynamic indices like Eadyn could provide further insights into optimizing fluid management strategies in elderly patients.

The limitations of this study must be acknowledged. First, direct intra-abdominal pressure measurements were not performed, which might have provided additional insights into the hemodynamic effects of prone positioning. Second, the single-center nature of the study and its relatively small sample size limit the generalizability of the findings. Third, objective evaluation parameters for vascular stiffness and arterial characteristics in the elderly patients included in the study (e.g., arterial stiffness index or pulse wave velocity measurements) were not incorporated [28]. Such data could have clarified the relationship between arterial properties and fluid responsiveness more effectively.

## 5. Conclusions

In conclusion, this study assessed the predictive ability of dynamic arterial elastance (Eadyn) for fluid responsiveness in elderly patients undergoing spine surgery in the prone position. Eadyn showed limited predictive value, while stroke volume variation (SVV), pulse pressure variation (PPV), and net arterial compliance (C) demonstrated better reliability, with SVV being the most accurate. These findings highlight the importance of using established dynamic parameters and exploring alternative markers like net arterial compliance to optimize fluid management in high-risk populations.

## Figures and Tables

**Table 1:** Patient characteristics (*n* = 37).

	Responders (*n* = 15)	Non-Responders (*n* = 22)
Gender (Female/Male)	8/7	16/6
Age, years	77 ± 6	76 ± 4
Weight, kg	59 ± 15	58 ± 10
Ideal body weight, kg	52 ± 9	48 ± 10
Height, cm	157 ± 9	153 ± 9
BMI	24 ± 5	25 ± 3
ASA PS (II/III)	5/10	7/15
Ventilator settings		
Tidal volume, mL/kg ideal body weight	427 ± 75	406 ± 68
Total PEEP, cmH_2_O	15 ± 2	16 ± 2
FiO_2_, %	60	60
SpO_2_, %	99 [98–100]	99 [98–100]
Operation type, *n*		
Discectomy	7	14
Laminectomy	6	2
Posterior fusion	2	6

All data are presented as mean ± SD, median [IQR] or number. Abbreviations: ASA PS, American Society of anesthesiologists physical status.

**Table 2:** Hemodynamic parameters in responder and non-responder patients to fluid loading (crystalloid 500 mL).

	Pre-Loading	Post-Loading	*p*-Value	Mean Diff.
Mean arterial pressure (mmHg)				
Responder	70 ± 9 ^a^	85 ± 10 ^ab^	<0.0001	14.53
Non-responder	80 ± 12	76 ± 12	0.0512	−4.27
Intergroup P	0.0092	0.0301		
Heart rate, beats/min				
Responder	72 ± 10	64 ± 6 ^b^	0.0023	−7.93
Non-responder	76 ± 12	66 ± 10 ^b^	<0.0001	−10.22
Intergroup P	0.359	0.6381		
Pulse pressure, mmHg				
Responder	41 ± 18	51 ± 13 ^b^	0.0088	10.07
Non-responder	60 ± 16	51 ± 12 ^b^	0.0021	−8.82
Intergroup P	0.655	0.979		
Cardiac output, L/min				
Responder	2.6 ± 0.8	3.1 ± 0.6 ^b^	0.0089	0.46
Non-responder	2.9 ± 0.6	3.2 ± 0.6 ^b^	0.0030	0.34
Intergroup P	0.359	0.6381		
Stroke volume (mL)				
Responder	37 ± 9	49 ± 9 ^b^	<0.0001	10.95
Non-responder	39 ± 11	50 ± 12 ^b^	<0.0001	12.00
Intergroup P	0.5629	0.7839		
Stroke volume Index (mL/m^2^)				
Responder	22 ± 5	30 ± 5 ^b^	<0.0001	8.00
Non-responder	24 ± 5	31 ± 7 ^b^	<0.0001	8.00
Intergroup P	0.2794	0.5428		
PPV (%)				
Responder	20 ± 3 ^a^	11 ± 4 ^b^	<0.0001	−8.80
Non-responder	15 ± 7	9 ± 5 ^b^	0.0002	−5.59
Intergroup P	0.0187	0.3003		
SVV (%)				
Responder	17 ± 4 ^a^	12 ± 5 ^b^	0.0023	−4.87
Non-responder	12 ± 5	9 ± 4 ^b^	0.0002	−2.95
Intergroup P	0.0030	0.0719		

All data are presented as means ± standard deviations. ^a^ *p*-value compared to non-responders using a *t*-test. ^b^ *p*-value compared to the pre-loading phase using a paired *t*-test. Abbreviations: PPV, pulse pressure variation; SVV, stroke volume variation.

**Table 3:** Effects of volume expansion on arterial tone parameters.

Parameter	Pre-Loading	Post-Loading	*p*-Value
Ea_dyn_			
Responder	1.2 ± 0.3	1.0 ± 0.4	0.1540
Non-responder	1.2 ± 0.4	1.0 ± 0.4 ^b^	0.0321
Intergroup P	0.9141	0.8707	
Ea, mmHg/mL			
Responder	2.6 ± 1.0	2.2 ± 0.7	0.0650
Non-responder	3.2 ± 1.3	2.2 ± 0.8 ^b^	<0.0001
Intergroup P	0.1222	0.9039	
C, mL/mmHg			
Responder	1.02 ± 0.40 ^a^	1.01 ± 0.28	0.9115
Non-responder	0.70 ± 0.29	1.04 ± 0.38 ^b^	<0.0001
Intergroup P	0.0076	0.8413	
SVR, dyn·s·cm^−5^			
Responder	2213 ± 818	2172 ± 587	0.7661
Non-responder	2426 ± 754	2032 ± 579 ^b^	0.0004
Intergroup P	0.4187	0.4777	

All data are presented as means ± standard deviations. ^a^ *p*-value compared to non-responders using a *t*-test. ^b^ *p*-value compared to the pre-loading phase using a paired *t*-test. Abbreviations: Ea_dyn_, dynamic arterial elastance; Ea, effective arterial elastance = pulse pressure/stroke volume; C, net arterial compliance; SVR, systemic vascular resistance.

## Data Availability

The data supporting the findings of this study are not publicly available but can be obtained from the principal investigator upon reasonable request.

## Supplementary Materials

The following supporting information can be downloaded at: https://www.mdpi.com/article/10.3390/jcm14041247/s1, Figure S1: study flow chart.

## Abbreviations

AUC	Area Under the Curve
ASA	American Society of Anesthesiologists
BIS	Bispectral Index
BMI	Body Mass Index
C	Net Arterial Compliance
CO	Cardiac Output
Ea	Effective Arterial Elastance
Eadyn	Dynamic Arterial Elastance (ratio of PPV to SVV)
HR	Heart Rate
KBSMC	Kangbuk Samsung Medical Center
LVEF	Left Ventricular Ejection Fraction
MAP	Mean Arterial Pressure
PPV	Pulse Pressure Variation
ROC	Receiver Operating Characteristic
SAP	Systolic Arterial Pressure
SV	Stroke Volume
SVI	Stroke Volume Index
SVR	Systemic Vascular Resistance
SVV	Stroke Volume Variation
TOF	Train-of-Four
