# Supplementary figures and images for: Dynamic Arterial Elastance as a Predictor of Intraoperative Fluid Responsiveness in Elderly Patient over 70 Years of Age Undergoing Spine Surgery in the Prone Position Under General Anesthesia: A Validation Study
Source: J Clin Med. 2025 Feb 13;14(4):1247. doi: 10.3390/jcm14041247 (PMC11856236; doi:10.3390/jcm14041247)

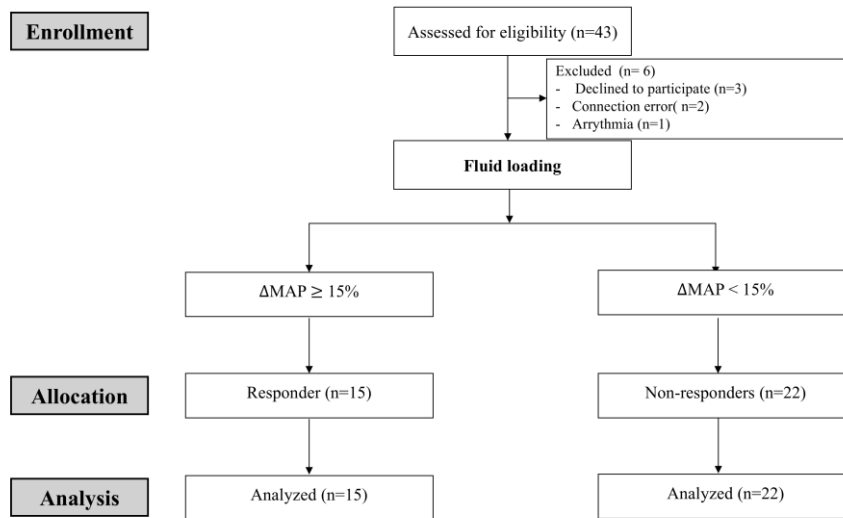

**Figure S1.** Study flow Chart.

Supplement: Supplementary file 1 [file jcm-14-01247-s001.zip › jcm-3423021-supplementary.pdf]
